# Outcomes of Road Traffic Accidents Before and After the Implementation of a Seat Belt Detection System: A Comparative Retrospective Study in Riyadh

**DOI:** 10.7759/cureus.27298

**Published:** 2022-07-26

**Authors:** Ibrahim Al Babtain, Aljawharah Alabdulkarim, Ghadah Alquwaiee, Shikah Alsuwaid, Eythar Alrushid, Maram Albalawi

**Affiliations:** 1 General Surgery, King Abdulaziz Medical City Riyadh, Riyadh, SAU; 2 Medicine and Surgery, King Saud Bin Abdulaziz University for Health Sciences College of Medicine, Riyadh, SAU; 3 Biostatistics, King Abdullah International Medical Research Center, Riyadh, SAU

**Keywords:** icu, ejection, gcs, iss, detection system, trauma, seatbelt, mortality, road traffic accidents

## Abstract

Introduction

Road traffic accidents (RTAs) are considered a major cause of death in Saudi Arabia. As seat belt compliance provides significant safety among drivers, a camera detection system has been implemented in March 2018 to enforce seat belt utilization, which can decrease the severity of road traffic injuries. There are no previous studies in the country that have assessed the effectiveness of a seat belt camera detection system on the severity of RTA-related injuries.

Methods

A retrospective cohort study was conducted at King Abdulaziz Medical Trauma Center in Riyadh, Saudi Arabia. The study included 688 adult patients who were involved in RTAs from the period of March 2016 to March 2020. A data extraction form included sociodemographics, clinical variables, and outcome measures. The data were analyzed using Statistical Analysis Software (SAS) to evaluate the primary outcome measures: mortality, ejection from the vehicle, ICU admissions, and severity measures (injury severity score (ISS) and Glasgow Coma Scale (GCS)) before and after the implementation of seat belt detection system. Associations of the outcome measures in the pre-implementation and the post-implementation periods' seat belt detection were assessed using regression tests.

Results

There was no significant difference in the mean age between the pre-implementation and post-implementation periods of the seat belt detection system (31.39 years and 32.57 years, respectively). All of the outcome measures have improved following the implementation of the seat belt detection system. Mortality and ejection rates decreased significantly with 58% lower risk of death (OR= 0.42; 95% CI= 0.2,0.8) and 37% lower risk of ejection (OR= 0.63; 95% CI= 0.42,0.94). ICU admissions showed a slight decline in the post-implementation period compared to the pre-implementation period (30.37% vs. 31.37, p<0.7764). Severity measures (ISS and GCS) were slightly improved in the post-implementation period. Head and neck injuries were dominant in the pre-implementation period, and chest injuries were the most common body injuries after the implementation.

Conclusion

This study highlights the direct association between compliance with seat belt use and the primary outcome measures among patients who survived a road traffic accident. All of the outcome measures showed improvement in the post-implementation period, which indicates the effectiveness of the newly implemented seat belt detection system. These findings raise awareness to the public in regard to seat belt compliance.

## Introduction

Road traffic accidents (RTAs) contribute to high rates of morbidity and mortality globally [1]. RTAs are estimated to be the eighth leading cause of death with millions of individuals suffering from more serious injuries, and by 2030, it is predicted to be the seventh leading cause among all age groups [2]. In the Kingdom of Saudi Arabia (KSA), 4.7% of all mortalities are due to RTAs, which is shown to be higher than RTA fatalities in Australia and United Kingdom, moreover, in comparison to the United States, which reports one death out of 283 RTAs, KSA reports one death out of 32 RTAs [3-4]. These numbers, in addition to what has been mentioned above, demonstrate the heavy burden of RTAs on KSA’s healthcare system and economy.

Among the various factors that can lead to fatal RTAs, failing to use seat belts is considered significantly to be one of those risk factors [5]. A seat belt is defined as a safety strap designed in vehicles, which is used as a method of protection to sustain drivers and passengers during rides. Seat belts work by moving in coordination with the driver/passenger’s body in a way that prevents ejection during collisions and crashes [6]. Studies have proved that adhering to seat belt use extensively minimizes the extent of injuries that can result from RTAs. Using a seat belt leads to a decrease in the probability of severe injuries and mortalities by 40% to 60% [7]. Furthermore, among drivers and passengers, seat belts prevent them from being ejected from the car, as failing to wear a seat belt increases the chance of being ejected by 30 times compared to those who are adherent to seat belt use [8]. In addition, seat belts significantly contribute to the variation of the injury pattern of the belted versus the unbelted vehicle occupants. For example, head injuries predominate in unbelted compared to belted occupants [9].

With the huge burden that seat belt non-compliance has put on the Saudi healthcare system and economy, on March 5, 2018, the Saudi Ministry of Interior implemented a seat belt tracking system and a law which mandates wearing the seat belt whenever a person is driving. The tracking system uses a network of digital cameras connected to the National Information System (NIS), which is part of the ministry. Detection cameras were implanted in multiple locations, which automatically issue penalty tickets for seat belt non-compliance. According to Alghnam et al., seat belt compliance has drastically increased after the implementation of the detection system, making vehicle drivers six times more likely to use seat belts than before the intervention [10].

To the best of our knowledge, no previous studies have assessed the impact of the new seat belt detection system in KSA on patients’ outcomes, and little is known about whether the introduction of the system would have an impact on the severity of RTA-related injuries and mortality rates. As it is essential to utilize scientific tools to evaluate the effectiveness of such interventions, our study aims to compare the severity of injuries resulting from RTAs before and after the implementation of a seat belt detection system in KSA at a Saudi Arabian level one trauma hospital. Additionally, the study will reflect the efficacy of the system.

## Materials and methods

A retrospective cohort analysis was conducted at a level one trauma center in Riyadh, Saudi Arabia. The dataset used in this study was a part of a trauma registry system of the trauma center.* *In 2001, the trauma registry was established to plan an injury prevention program, as well as to monitor and facilitate the process of patient care. Patients who are included in the registry should meet at least one of the following: (1) presenting to the ED after an acute injury requiring admission to the hospital ward or intensive care unit; (2) indirect admission (patient discharged from ED and asked to return later); (3) transfer to surgery from the ED; or (4) declared dead after initial evaluation in the ED or before arrival. Patients’ demographic, physiologic, anatomic, and outcome variables are gathered in a structured checklist, filled and completed by a nurse, and then a trained research coordinator enters the information into the registry system.

To be included in our study, patients should have been involved in an RTA as drivers and transferred to and admitted to KAMC between March 2016 and March 2020. All patients who were below 18 years, front seat passengers or other passengers in the vehicle, and those who were dead on scene or dead on arrival were excluded.

The main objective of the study was to create an indicator variable to compare the periods before and after the implementation of the seat belt detection system in KSA, which was officially initiated in March 2018. The data were extracted two years before and two years after the implementation. The period March 2016 to March 2018 is the pre-implementation period, and the period March 2018 to March 2020 is the post-implementation period. The exact date of the implementation was March 5, 2018, so the pre-implementation and post-implementation periods were categorized according to this date.

The data were extracted from the registry of all cases by the co-authors, and supervised by a consultant of general and trauma surgery. The data extracted included patient’s demographic information (age at the time of RTA and gender), seat belt use, administration status, including injury severity score (ISS), Glasgow coma scale (GCS), ejection, loss of consciousness (LOC), and trauma team activation, in addition to the body region of injury, including head and neck, chest, abdomen, and upper and lower extremities. Finally, the patients’ treatment plans, including surgical intervention, patients' outcomes including length of hospital stay, ICU admission, cardiac arrest with resuscitation, and death were all included as well.

Outcome measures

This study focused on four primary outcome measures to assess the extent of the severity and whether the implementation of the seat belt detection system has had an impact on those outcomes. The primary outcomes included: Mortality, Ejection, ICU admissions, and Severity measures. Severity measures included the ISS and GCS. ISS is one of the gold standard tests to assess the anatomical severity of an injury with 0 and 75 as the lowest and highest possible scores, respectively, and higher scores indicate more severe injuries. ISS is derived from the abbreviated injury scale (AIS), which categorizes the body into six regions with a given score for each, and then the three most severely injured regions are squared and summed up to calculate ISS [11-12]. GCS is a scoring tool that assesses post-trauma physiological impairment by measuring the level of consciousness, looking into the eye, motor, and verbal responses, and then calculating the scores of each with a final score ranging from 3-15 and higher scores indicating a better physiological status. The secondary outcomes included injured body regions (head and neck, chest, abdomen, upper and lower extremity), loss of consciousness, trauma team activation, hospital length of stay, surgical intervention, and cardiac arrest with resuscitation. These primary and secondary outcome measures (dependent variables) were investigated and compared between the pre-implementation and the post-implementation periods as independent variables [13].

Statistical analysis

Categorical variables were presented as frequency and percentage while continuous variables were presented as mean and standard deviation (SD). The chi-square test was used to assess the association between categorical variables, whereas the Wilcoxon two-sample test was used for two-level continuous variables. The findings were considered statistically significant if p-value <0.05. The logistic regression test was used to assess the relationship between the dependent and independent variables. All data were analyzed using the statistical program SAS version 9.4 (SAS Institute Inc., Cary, NC). This study was reviewed and approved by the Institutional Review Board (IRB) at King Abdullah International Medical Research center (KAIMRC), with IRB no. NRC21R/184/06.

## Results

A total of 688 patients who were victims of road traffic accidents (RTAs) were reviewed and included in this study. Of these 688 patients, 306 had an RTA before the law on seat belts was implemented (March 5, 2016 - March 4, 2018) and 382 after the implementation (March 5, 2018 - March 5, 2020). Table 1 shows the patients' demographic data and seat belt status. The mean age of patients was 31.39 years and 32.57 years in the pre-implementation and post-implementation periods, respectively, and there was no significant difference between the two periods (p<0.194). Out of these 688 patients, a total of 673 (97.82%) were males and 15 (2.18%) were females. The gender showed a significant difference between the numbers of males and females during the post-implementation period, with a total of 368 (96.34%) and 14 (3.66%), respectively (p<0.0092). During the post-implementation period, out of these 382 victims who were involved in RTAs, 52 (13.61%) patients were restrained and 195 (51.05%) were unrestrained, and the seat belt status was missing for a total of 135 (35.34%) patients.

**Table 1:** Demographic data and seat belt status *Chi-square test; ^Wilcoxon two-sample test SD: standard deviation

Variable	Pre-implementation N=306 (%)	Post-implementation N=382 (%)	All Patients N=688	P-value
Age (mean, SD)	31.39 (14.60)	32.57 (14.85)		0.194^
Gender				0.0029*
Female	1 (0.33)	14 (3.66)	15	
Male	305 (99.67)	368 (96.34)	673	
Seat belt				< .0001*
Restrained	11 (3.59)	52 (13.61)	63	
Unrestrained	201 (65.69)	195 (51.05)	396	
Not mentioned	94 (30.72)	135 (35.34)	229	

**Figure 1:**
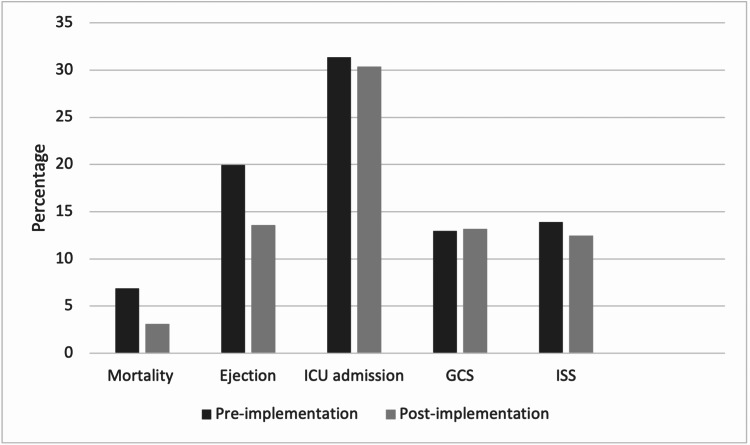
Descriptive analysis (percentage) of the outcome measures between pre-implementation and post-implementation periods CU: intensive care unit, GCS: Glasgow Coma Scale, ISS: injury severity score

Regarding the primary outcome measures, as shown in Table 2, mortality rates declined significantly from 6.8% to 3.1% (P=0.002). In regards to whether or not the victim was ejected from the car, there was a slight decline in the number of ejections during the post-implementation period (n= 52; 13.61%) compared to the pre-implementation period (n=61; 19.93%) (p<0.0261). Injury severity measurements, including ISS and GCS, as well as ICU admissions, showed no significant difference between the two periods, despite slightly improved severity scores and admission rates in the post-implementation period. These results show that all of the primary outcome measures have improved when compared between the pre-implementation and post-implementation periods (Figure 1). Concerning the secondary outcomes, the most common body region affected before the implementation was the head and neck regions (43.13%), and the most common region affected after the implementation was the chest (47.37%). There was no significant difference in head and neck injuries as well as in the lower extremity before and after the implementation periods. However, a significant difference in the number of chest, abdomen, and upper extremity injuries between the two periods has been confirmed (P=0.03, P=0.03, P=0.01, respectively). There was no difference in the proportion of loss of consciousness, trauma team activation, or hospital length of stay in the pre-implementation and post-implementation periods of the seat belt detection system. In addition to that, the number of patients undergoing major surgical intervention following RTAs also showed no significant difference in the period before and after the implementation. The occurrence of a cardiac arrest with resuscitation slightly declined in the post-implementation period.

**Table 2:** Descriptive analysis of the primary and secondary outcome measures documented in KAMC by admission periods: pre-implementation (March 5, 2016 - March 4, 2018) and post-implementation (March 5, 2018 - March 5, 2020) *Chi-square test; ^Wilcoxon two-sample test KAMC: King Abdulaziz Medical City, SD: standard deviation, ICU: intensive care unit, GCS: Glasgow Coma Scale, ISS: injury severity score

Variable	Pre-implementation N=306 (%)	Post-implementation N=382 (%)	All Patients N=688	P-value
Primary outcome measures				
Mortality	21 (6.86)	12 (3.14)	33	0.0232*
Ejection	61 (19.93)	52 (13.61)	113	0.0261*
ICU admission	96 (31.37)	116 (30.37)	212	0.7764*
GCS (mean, SD)	12.95 (3.64)	13.18 (3.49)		0.202^
ISS (mean, SD)	13.89 (11.64)	12.49 (10.27)		0.092^
Secondary outcome measures				
Body region				
Head and neck	132 (43.14)	154 (40.31)	286	0.4553*
Chest	121 (39.54)	181 (47.38)	302	0.0395*
Abdomen	59 (19.28)	100 (26.18)	159	0.0330*
Upper extremity	80 (26.14)	132 (34.55)	212	0.0176*
Lower extremity	121 (39.54)	153 (40.05)	274	0.8920*
Loss of consciousness	85 (27.78)	90 (23.56)	175	0.2068*
Trauma team activation	110 (35.95)	132 (34.55)	242	0.7038*
Length of stay (mean, SD)	6.34 (6.09)	7.06 (6.50)	375	0.106^
Surgery	157 (64.61)	218 (72.67)	375	0.0941*
Cardiac arrest with resuscitation	19 (6.21)	7 (1.83)	26	0.0028*

Regression analysis was conducted to assess the improvement in the outcome measures (mortality, ejection, and ICU admissions) following the implementation. In Table 3, the logistic regression model confirmed an improvement in mortality, ejection, and ICU admissions with 58% and 37% lower risk in the post-implementation period, respectively. ICU admission showed only a slight improvement after the implementation with a 5% lower risk.

**Table 3:** Logistic regression analysis of the categorical outcome measures (mortality, ejection, and ICU admissions) * 95% Confidence Interval ICU: intensive care unit

Variables	Logistic regression (odds ratio for mortality)	Logistic regression (odds ratio for ejection)	Logistic regression (odds ratio ICU admission)
Pre-Implementation	Reference	Reference	Reference
Post-Implementation	0.425 (0.203, 0.889)*	0.633 (0.422, 0.949)*	0.954 (0.689, 1.321)*

## Discussion

RTAs represent a major global public health concern because of their increasing occurrence, related mortality, morbidity, and social and financial consequences [14]. In SA, over 80% of all trauma admissions in KAMC account for RTAs. As most of these trauma cases could be prevented by the government’s efforts to decrease the number of RTAs using the newly implemented seat belt detection system, we found in our study that there are variable results in the outcome measures (mortality, ejection, ICU admissions, ISS, and GCS) among patients after the implementation of the seat belt detection system in Riyadh. The outcome of this study showed that the investment in traffic and drivers’ safety interventions is not wasted, and further improvements in these facilities can maintain health resources and decrease the burden on public health.

In our study, we found low compliance in wearing seat belts despite the increase in awareness and efforts that were established to enforce the compliance of seat belt wearing in the country. Various studies have reported numerous different results. The low seat belt compliance here is consistent with two previous studies that reported the overall rate of seat belt use as 27.85% and 34%, respectively (Alghnam et al., [5,15]). In comparison to that, a study that was conducted in Italy reported 51.8% use of seat belts [16]. A possible explanation for our results is the fact that the seat belt detection system in the country is newly implemented and our data were explored two years prior to the implementation. We expect that not everyone is compliant with the law, as it may not represent sufficient time to change the drivers’ behavior, and efforts should be made to adopt such a change. Of note, some of our patient's compliance in regards to wearing a seat belt was not identified in the registry system, and this could be due to a lack of proper documentation, and further studies and efforts are needed to address the importance of documenting that.

In our study, there was a significant reduction in mortality rate and occurrence of cardiac arrest with resuscitation following the implementation of the system. This is consistent with a previous study that showed a similar decline in death rates among those who were belted vs unbelted victims [1]. This reduction in the number of deaths can potentially be explained by the increase in the number of people who were adherent to seat belt use after the system has been implemented, which, as a result, has led to less traumatic, less severe RTA injuries and fewer body regions involved.

With regards to body region injuries, chest and abdomen injuries have been noticed in this study to be higher in the post-implementation period. This is consistent with other studies in which they found that chest injuries (including pneumothorax, hemothorax, rib, and sternal fractures) were more common in belted patients [1,9,17]. However, Porter et al. (1998) noted that abdominal injuries are equal in both groups [9]. These findings could be explained by the physiological impact of the seat belt that causes increased pressure on the chest and abdominal area. On the other hand, lower extremity injuries showed no significant difference in the pre-implementation and post-implementation periods. This finding is consistent with previous studies, as they reported no statistically significant difference in the risk of lower limb injury in belted versus non-belted victims since the lower part of the body is unprotected by the seat belt, and the way of wearing the seat belt by the victims could contribute to these findings and increase the unprotection of lower extremities [7,9].

A slight increase in the scores of GCS was seen in our study after the implementation of the system. The mean GCS was 12.95 and 13.18 before and after the implementation, respectively. The proportion of patients with a GCS of 8 and below decreased slightly after the implementation of the system. This is consistent with another study that found GCS to be 8 or below in the unrestrained group of patients than in the restrained group [1]. This increase in GCS scores and the decline in loss of consciousness can be attributed to the fact that the majority of restrained victims did not suffer from a head injury, which is the main cause of the drop in GCS and the level of consciousness. In addition, our study showed declined ISS after the implementation, which is similarly consistent with the same study that found lower ISS among the restrained group of patients [1]. Similar to previous studies, our results found that the rates of ICU admissions and surgical interventions were slightly lower in the post-implementation period [1]. However, only a slight difference was noted in our study, and one explanation for this is that the group of patients in the post-implementation period could potentially be involved in more severe and traumatic accidents that may have led to a worse presentation requiring ICU admission and major interventions. Also, the total number of RTAs in the post-implementation period was higher in comparison with the pre-implementation period.

Seat belt utilization reduces the chance of a driver being ejected from the car. In our study, ejection was reduced in patients in the post-implementation versus pre-implementation periods. This is similar to other studies that showed a 38% reduction rate in patines who were restrained, and another study that reported 62% of unrestrained drivers ejected from the vehicle [18-19].

Contrary to our study, it has been reported in several studies that there were more belted females who are involved in RTAs than belted males [1,20]. However, in our study, there was a significant difference in gender, and belted females involved in the RTAs were lower than belted males. This contrast could be explained by the fact that women in KSA were banned from driving until June 2018 when the ban was officially lifted, and women were allowed to drive for the first time ever. Since our study data included RTAs that occurred from 2016-2020, this difference in gender compliance to seat belt use could be attributed to the low number of females (n=15) compared to males (n= 673) that were included in the study, which is mainly due to the previous driving ban, and even with the new guidelines, the number of females driving in KSA is still relatively low.

This study is not without limitations. First, and because it is a retrospective study, not all data were available in the registry system, including some participants who did not have clear specifications of their injuries and those who were dead on the scene. Second, seat belt information was not all documented in regards to seat belt use, the data available was limited, and we were not able to determine whether those who were using seat belts are using it appropriately. Also, since detection cameras are not implanted everywhere in Riyadh, we cannot determine whether those RTAs occurred in areas with available detection cameras, as they are only limited to the major roads and highways, therefore drivers are unlikely to get fined a violation fee in some areas. In addition, the cameras can only detect the driver's seat belt use, but cannot detect the front seat or the rear seat passengers' seat belts. Lastly, the data in this study are considered somehow limited, taking into consideration that the paper covered only two years after the implementation of the system, and the sample was taken from one city and one trauma center.

This study has several strengths. It is the first study in Saudi Arabia to compare the severity of injuries resulting from RTAs before and after the implementation of the seat belt detection system. In addition to that, the sample size that was studied in this paper included patients from military staff, healthcare workers, students, and other members from the community, which represents the diversity of backgrounds that somehow represent the Saudi population. Furthermore, our findings may support Saudi Vision 2030, which is a national landmark plan that aims to enhance the population's well-being and decrease disabilities [21]. The new detection system is one of the supported programs by Saudi Vision 2030, and it aims to increase awareness of seat belt wearing and invest in preventive strategies as one of its many goals. Our study may encourage further studies to evaluate new interventions and measurements, and explore the causes and possible solutions to increase awareness and education in order to improve drivers’ compliance, maintain public health, and increase road safety.

## Conclusions

In summary, the overall rate of seat belt use remains low in our country compared to developed countries. This raises the importance of increasing awareness among the public in regard to seat belt compliance and reducing road traffic accidents. Further investment in public health strategies to improve prevention methods needs to be addressed. We also found, in this study, that reduced rates of mortality and cardiac arrests in addition to an improvement in GCS and ISS scores following the implementation of the seat belt detection system. This indicates that compliance with seat belts has improved patient outcomes and road safety, and that seat belt use has a major role in protecting victims during RTAs. Therefore, seat belt use while driving should continue to be observed, and further enforcement efforts are required to maintain and improve public health and traffic safety.
